# KODAMA: an R package for knowledge discovery and data mining

**DOI:** 10.1093/bioinformatics/btw705

**Published:** 2016-11-30

**Authors:** Stefano Cacciatore, Leonardo Tenori, Claudio Luchinat, Phillip R Bennett, David A MacIntyre

**Affiliations:** 1 Institute of Reproductive and Developmental Biology, Imperial College London, London, UK; 2 Department of Clinical and Experimental Medicine, University of Florence, Florence, Italy; 3 Centro Risonanze Magnetiche, University of Florence, Florence, Italy

## Abstract

**Summary:**

KODAMA, a novel learning algorithm for unsupervised feature extraction, is specifically designed for analysing noisy and high-dimensional datasets. Here we present an R package of the algorithm with additional functions that allow improved interpretation of high-dimensional data. The package requires no additional software and runs on all major platforms.

**Availability and Implementation:**

KODAMA is freely available from the R archive CRAN (http://cran.r-project.org). The software is distributed under the GNU General Public License (version 3 or later).

**Supplementary information:**

Supplementary data are available at *Bioinformatics* online.

## 1 Introduction

Knowledge Discovery and Data Mining is an interdisciplinary area focusing upon methodologies for extracting useful knowledge from complex data. With the explosive growth of high-throughput experimental data, data-based solutions are increasingly crucial. We recently published KODAMA, a novel learning algorithm for unsupervised feature extraction, specifically designed for analysing noisy and high-dimensional datasets (Cacciatore *et al.*, 2014). This versatile method has been successfully applied to a wide range of disciplines including genomics (Cacciatore *et al.*, 2014) and metabolomics (Priolo *et al.*, 2014) and has even been used in the analysis of hyper-spectral images (Cao *et al.*, 2016). Here, we present for the first time the *KODAMA* package developed for use in the R programming environment.

The core of the algorithm consists of two main parts. The first step involves random assignment of each sample to a different class. In the second step, the cross-validated accuracy is maximized by an iterative procedure by swapping the class labels (no *a priori* information is needed). The cross-validated accuracy can be calculated by using any supervized classifier. In the current version of KODAMA, two classifiers are implemented: *k*-Nearest Neighbors (*k*NN) and Partial Least Squares (PLS)—Discriminant Analysis (DA). The iterative procedure used in KODAMA leads to suboptimal solutions and must be repeated (100 as default) to average the effects owing to randomness. External class information can be integrated in KODAMA before performing the iterative procedure thereby supporting a semi-supervized approach for highlighting otherwise hidden features of interest. After each run of the procedure, a classification vector with high cross-validated accuracy is obtained. KODAMA subsequently collects and processes these results by constructing a dissimilarity matrix to provide a holistic view of the data while maintaining their intrinsic structure.

Here, we show that KODAMA demonstrates high capacity to detect different underlying relationships in experimental datasets including patient phenotypes (Cacciatore *et al.*, 2014; Priolo *et al.*, 2014). We also introduce the possibility of using KODOMA to correlate extracted features describing phenotype with accompanying metadata.

## 2 Methods

The revised *KODAMA* package includes improvements in the implementation of the code and seven major new functions: *pls.kodama*, *knn.kodama*, *pls.double.cv*, *knn.double.cv, k.test*, *loads* and *mcplot*.

The package is computationally efficient with the workhorse functions written in C ++ using Rcpp (Eddelbuettel, 2011), RcppArmadillo (Eddelbuettel and Sanderson, 2014) and integrating the Approximate Nearest Neighbour Searching (ANN) library (Arya *et al.*, 1998). Functions coded in C ++ include *k*NN (*knn.kodama*) and PLS-DA (*pls.kodama*) classifiers and the iterative procedure of KODAMA. The *pls.double.cv* and *knn.double.cv* functions perform double cross-validation procedures using PLS-DA or *k*NN as classifiers, respectively (Bertini *et al.*, 2012). The internal parameter (i.e. number of components or *k*) is optimized by maximising the cross-validated coefficient of determination (Q^2^y) obtained by an inner cross-validation on the training sets.

The *loads* function can be used to extract the variable ranking. After each maximization of the cross-validated accuracy the final label set is used to calculate the loadings of PLS-DA or the logarithm of the *P*-value from the Kruskal-Wallis Rank Sums test. The output of *LOADS* function is the average of these values for each variable of the dataset. The highest values indicate the most important variables.

The *k.test* function performs a statistical test to assess association between the KODAMA output and any additional related parameters such as clinical metadata. The coefficient of determination (*R*^2^) is used to assess the proportion of the variance in the dependent variable (KODAMA output) that is predictable from the independent variable (e.g. clinical parameter) and can thus be used as a measure of the goodness of fit (Cameron *et al.*, 1997). A permutation test is performed by randomly sampling the value of the labels to estimate the significance of the observed association.

The *mcplot* function is now included as a diagnostic solution of the iterative process for maximization of cross-validated accuracy. This function visualizes the values of accuracy step-by-step through each separate iterative process.

The Shannon Entropy (Shannon, 1948), is now implemented as output of the *KODAMA* function and can be used as a measure of unpredictability of information content to select the optimal classifier and its relative parameter.

## 3 Results

**Fig. 1.**
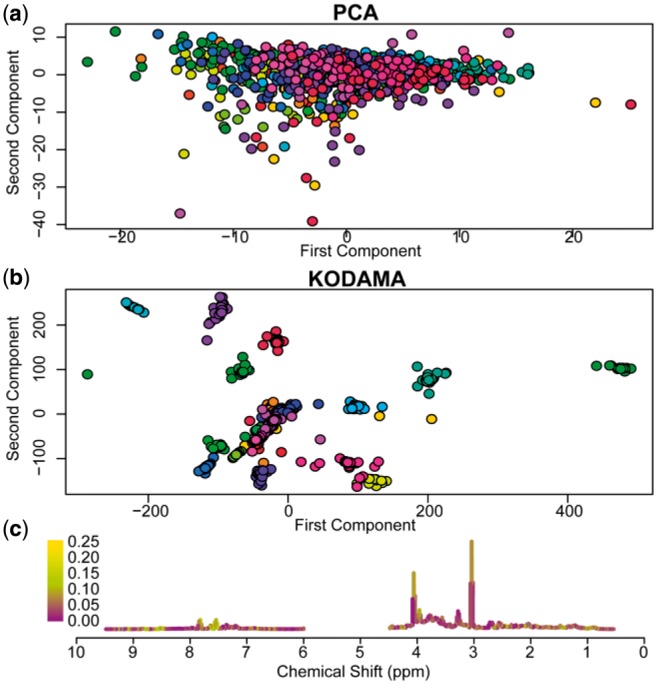
(**a**) PCA and (**b**) KODAMA of MetRef dataset. Color coding indicates samples from the same donor. (**c**) Average NMR spectrum of MetRef dataset. Color-code represents the output of the LOADS function. The spectral features with the highest contribution to the spatial separation observed in the KODAMA output are represented in yellow

To demonstrate briefly the performance of the *KODAMA* package, we used the *MetRef* dataset (included in this package), a collection of 873 nuclear magnetic resonance spectra of urine samples from a cohort of 22 healthy donors (11 male and 11 female). Figure 1 shows a comparison between KODAMA and Principal Component Analysis (PCA), an unsupervised method widely used in metabolic profiling (Aimetti *et al.*, 2012; MacIntyre *et al.*, 2010). As can be observed in Figure 1a, PCA provides comparatively poor description of the underlying variation in metabolic profiles of urine collected from healthy individuals. In contrast, KODAMA (Fig. 1b) permits identification of the underlying patient-specific signature of the urine metabolome in an unsupervised fashion. This important biologically relevant information would have been otherwise lost using PCA. The script for generating Figure 1a, b is included in Supplementary material. Figure 1c highlights spectral features most responsible for separation of patient samples obtained with the *loads* function. Further analysis using the *k.test* function shows a statistically significant association between the KODAMA output with clinical metadata including donor (*P *<* *0.0001) and gender (*P *<* *0.0001).

## 4 Summary and outlook

KODAMA represents a valuable tool for performing feature extraction on noisy and high-dimensional datasets. Addition functions facilitate the identification of key features associated with the generated output and are easily interpretable for the user. The K-test permits the identification of significant associations between the KODAMA output and related information.

## Supplementary Material

Supplementary Data
