# Supplementary material for: KODAMA: an R package for knowledge discovery and data mining
Source: Bioinformatics. 2016 Nov 30;33(4):621–3. doi: 10.1093/bioinformatics/btw705 (PMC5408808; doi:10.1093/bioinformatics/btw705)
Supplement: Supplementary Data [file btw705_supp.docx]

**Supplementary data for “KODAMA: an R package for knowledge discovery and data mining”. Cacciatore *et al.*, 2016**

**Supplementary Methods**

Here we present the script required for generating Figure 1a-b presented in the article using the *MetRef* data set (included in this package). It is a collection of 873 nuclear magnetic resonance spectra of urine samples from a cohort of 22 healthy donors (11 male and 11 female) where each donor provided approximately 40 urine samples over a 2 month time course.

Firstly, the MetRef data set is pre-processed. This involves removing zero values from the *MetRef* data set matrix and correcting for variations in urine concentration using Probabilistic Quotient Normalization (Dieterle *et al.*, 2006). The data are then mean-centered and unit variance scaled.

> data(MetRef)

> # Zero values are removed.

> MetRef$data=MetRef$data[,-which(colSums(MetRef$data)==0)]

> # Normalization of the data set with the Probabilistic Quotient Normalization method.

> MetRef$data=normalization(MetRef$data)$newXtrain

> # Centers the mean to zero and scales data by dividing each variable by the variance.

> MetRef$data=scaling(MetRef$data)$newXtrain

After pre-processing, KODAMA is then run with different input settings. The two different classifiers are tested: *k*‑Nearest Neighbors (*k*NN) and Partial Least Squares (PLS) – Discriminant Analysis (DA). The *k*NN classifier is set with *k*=2,3,5,10,15 and 20. The PLS-DA classifier is set with number of components = 2,5,10,20,50 and 100.

> kk1=KODAMA2(MetRef$data,FUN = "PLS-DA",f.par = 2)

> kk2=KODAMA2(MetRef$data,FUN = "PLS-DA",f.par = 5)

> kk3=KODAMA2(MetRef$data,FUN = "PLS-DA",f.par = 10)

> kk4=KODAMA2(MetRef$data,FUN = "PLS-DA",f.par = 20)

> kk5=KODAMA2(MetRef$data,FUN = "PLS-DA",f.par = 50)

> kk6=KODAMA2(MetRef$data,FUN = "PLS-DA",f.par = 100)

> kk7=KODAMA2(MetRef$data,FUN = "KNN",f.par = 2)

> kk8=KODAMA2(MetRef$data,FUN = "KNN",f.par = 3)

> kk9=KODAMA2(MetRef$data,FUN = "KNN",f.par = 5)

> kk10=KODAMA2(MetRef$data,FUN = "KNN",f.par = 10)

> kk11=KODAMA2(MetRef$data,FUN = "KNN",f.par = 15)

> kk12=KODAMA2(MetRef$data,FUN = "KNN",f.par = 20)

The Shannon Entropy (*H*) (Shannon, 1948), a measure of unpredictability of information content, can then be used to select the optimal classifier and its relative parameter. *H* is calculated using the values of the KODAMA proximity matrix and is given by:

$$H=\sum_{i} \sum_{j} v_{ij}\times\log v_{ij}$$

where *v_ij_* is the proximity value between the sample *i* and the sample *j*, divided by the sum of overall proximities. Different classifiers can lead to solutions that represent of different views of the same dataset. Lowest value of *H* is used to select the best result solution.

> print(kk1$entropy)

[1] 13.493

> print(kk2$entropy)

[1] 13.508

> print(kk3$entropy)

[1] 13.496

> print(kk4$entropy)

[1] 13.444

> print(kk5$entropy)

[1] 13.245

> print(kk6$entropy)

[1] 13.093

> print(kk7$entropy)

[1] 13.460

> print(kk8$entropy)

[1] 13.428

> print(kk9$entropy)

[1] 13.425

> print(kk10$entropy)

[1] 13.420

> print(kk11$entropy)

[1] 13.426

> print(kk12$entropy)

[1] 13.450

With *H* = 13.093, KODAMA using PLS-DA (with 100 components) as classifier represents the best solution for the MetRef data set.

Principal Component Analysis (PCA) is showed for comparison purposes. Colour coding indicates samples from the same donor.

> pca=prcomp(MetRef$data)$x

> plot(pca,pch=21,bg=rainbow(25)[donor],xlab="First Component", ylab="Second Component")

The result of KODAMA with PLS-DA (100 components) as classifier is consequently showed. The KODAMA output saved in the variable *pp* is calculated by applying Sammon’s Non-Linear Mapping (Sammon 1969) to the KODAMA dissimilarity matrix.

> plot(kk6$pp,pch=21,bg=rainbow(25)[donor],xlab="First Component", ylab="Second Component")

The performance of both feature extraction methods is analysed by estimating the relative class overlap using the Davis-Bouldin Index (DBI) (Davies and Bouldin, 1979), a function of the ratio of the sum of within-cluster scatter to between-cluster separation, as implemented in the function DBIndex of the R library RDRToolbox. DBI is defined as:

$$DBI=\frac{1}{nc}\sum_{i=1,i\neq j}^{n} max\left[ \frac{\sigma_{i}+\sigma_{j}}{d(c_{i},c_{j})} \right]$$

where *nc* is the number of clusters, *σ_i_* is the average distance of all samples in cluster *i* to their cluster center *c_i_*, *σ_j_* is the average distance of all samples in cluster *j* to their cluster center *c_j_*, and *d(c_i_,c_j_)* is the distance of cluster centers *c_i_* and *c_j_*. Small values of DBI correspond to clusters that are compact, and whose centers are far away from each other.

> require(RDRToolbox)

> DBIndex(data = kk6$pp,labels = donor)

[1] 0.06047128

> DBIndex(data = pca, labels = donor)

[1] 0.592102

A DBI value of 0.06 indicates that the performance of KODAMA is significantly better than PCA (DBI=0.59).

The results of KODAMA are also validated by performing hierarchical clustering (Ward’s method) to the KODAMA dissimilarity matrix and comparing the results to hierarchical clustering (Ward’s method) performed on the Euclidean distance matrix. For both methods, a total of 22 clusters is imposed.

Firstly clustering based on KODAMA is performed as follows;

> clu1=cutree(hclust(as.dist(kk6$dissimilarity),method="ward.D"),22)

> tt=table(clu,MetRef$donor)

AD BG AF AG AH AI AO AP AR AS AT AU AW AX AZ BC BD BE BF BH BI BK

1 39 0 0 2 0 0 1 0 0 0 0 0 0 0 0 0 0 0 0 0 3 0

2 1 40 0 0 0 0 0 0 0 0 0 0 0 0 0 0 0 0 0 0 0 1

3 0 0 40 0 0 0 0 0 0 0 0 0 0 0 0 0 0 0 0 0 0 0

4 0 0 0 37 0 0 0 0 0 0 0 0 0 0 0 0 0 0 0 0 0 0

5 0 0 0 0 37 0 0 0 0 0 0 0 0 0 0 0 0 0 0 0 0 0

6 0 0 0 0 0 40 0 0 0 0 0 0 0 0 0 0 0 0 0 0 0 0

7 0 0 0 0 0 0 39 0 0 0 0 0 0 0 0 0 0 0 0 0 0 0

8 0 0 0 0 0 0 0 39 0 0 0 0 0 0 0 0 0 0 0 0 0 0

9 0 0 0 0 0 0 0 1 40 0 0 0 0 0 0 0 0 0 0 0 0 0

10 0 0 0 0 0 0 0 0 0 39 0 0 0 0 0 0 0 0 0 0 0 0

11 0 0 0 0 0 0 0 0 0 0 40 0 0 0 0 0 0 0 0 0 0 0

12 0 0 0 0 0 0 0 0 0 0 0 39 0 0 0 0 0 0 0 0 0 0

13 0 0 0 0 0 0 0 0 0 0 0 0 40 0 0 0 0 0 0 0 0 0

14 0 0 0 0 0 0 0 0 0 0 0 0 0 40 0 0 0 0 0 0 0 0

15 0 0 0 0 0 0 0 0 0 0 0 0 0 0 41 0 0 0 0 0 0 0

16 0 0 0 0 0 0 0 0 0 0 0 0 0 0 0 25 2 0 0 0 0 0

17 0 0 0 0 0 0 0 0 0 0 0 0 0 0 0 15 38 0 0 0 0 0

18 0 0 0 0 0 0 0 0 0 0 0 0 0 0 0 0 0 40 0 0 0 0

19 0 0 0 0 0 0 0 0 0 0 0 0 0 0 0 0 0 0 40 0 0 0

20 0 0 0 0 0 0 0 0 0 0 0 0 0 0 0 0 0 0 0 38 0 0

21 0 0 0 0 0 0 0 0 0 0 0 0 0 0 0 0 0 0 0 0 37 0

22 0 0 0 0 0 0 0 0 0 0 0 0 0 0 0 0 0 0 0 0 0 39

Next, hierarchical clustering is performed using the Euclidean distance matrix as described below;

> clu2=cutree(hclust(dist(MetRef$data),method="ward.D"),22)

> tt=table(clu,MetRef$donor)

AD BG AF AG AH AI AO AP AR AS AT AU AW AX AZ BC BD BE BF BH BI BK

1 7 6 0 1 1 1 0 2 0 3 3 0 5 0 1 8 10 0 8 2 0 1

2 0 15 0 24 0 2 0 0 0 2 0 0 0 0 0 18 3 0 0 0 0 2

3 1 1 28 1 1 0 28 0 0 0 0 0 0 0 0 1 0 0 1 26 0 4

4 0 0 0 0 3 0 0 0 0 0 1 0 0 0 0 0 0 0 0 0 0 1

5 1 2 5 2 24 1 0 0 0 1 4 2 0 0 0 3 3 2 0 1 1 0

6 3 0 0 0 0 25 0 0 0 0 0 0 0 0 0 0 0 0 0 0 0 0

7 1 0 1 3 0 2 6 1 0 1 0 0 1 0 0 1 0 0 4 0 0 3

8 0 1 0 2 1 0 0 25 0 0 0 0 1 2 0 4 16 8 0 3 0 3

9 0 0 0 1 0 4 0 1 33 0 0 0 0 0 0 0 0 0 0 0 0 0

10 1 6 0 1 1 0 1 7 0 30 0 0 0 0 0 0 0 0 24 0 0 23

11 0 4 3 1 1 0 1 3 0 0 30 3 0 8 0 1 0 0 0 3 8 0

12 0 0 0 0 0 0 0 0 0 0 0 28 0 0 0 0 0 0 0 0 0 0

13 0 0 0 0 0 0 0 0 0 0 0 0 24 0 0 0 0 0 0 0 0 0

14 2 0 1 0 0 0 1 1 4 1 0 2 0 27 0 1 2 3 0 1 0 0

15 0 2 0 0 0 0 0 0 0 1 0 0 0 0 29 0 0 0 0 0 0 0

16 0 0 0 0 1 0 0 0 3 0 0 3 0 0 0 1 0 0 0 0 0 0

17 3 0 2 1 2 4 1 0 0 0 0 0 8 3 8 0 5 0 0 0 0 0

18 0 0 0 0 0 0 0 0 0 0 0 0 0 0 0 0 0 26 0 1 0 0

19 0 1 0 0 1 0 0 0 0 0 0 0 0 0 0 0 0 0 3 0 0 2

20 0 0 0 0 1 0 1 0 0 0 1 1 1 0 0 0 1 1 0 1 3 0

21 20 2 0 0 0 1 1 0 0 0 1 0 0 0 3 2 0 0 0 0 25 1

22 1 0 0 2 0 0 0 0 0 0 0 0 0 0 0 0 0 0 0 0 3 0

Using the KODAMA dissimilarity matrix, only 3.0% of samples failed to be correctly clustered compared to >50% when Euclidean distance matrix is used.

The performance of the two different clustering approaches are also compared using the Adjusted Rand Index (ARI) (Hurbet and Arabie, 1985); a function that measures similarity between two classifications. ARI spans from -1 to 1; perfect agreement is scored 1, whereas 0 corresponds to a random partition. Negative values indicate less agreement than expected by chance. ARI is calculated using the function adjustedRandIndex from the R library mclust.

> require(mclust)

> adjustedRandIndex(donor,clu2)

[1] 0.316972

> adjustedRandIndex(donor,clu1)

[1] 0.9452274

The performance of the KODAMA approach (0.95) is superior to that of the classical Euclidean approach (0.32).

**References**

Davies,D.L. and Bouldin,D.W. (1979) A cluster separation measure. IEEE Trans Patter Anal Machine Intell, 1, 224-7.

Dieterle,F. *et al.* (2006) Probabilistic Quotient Normalization as Robust Method to Account for Diluition of Complex Biological Mixtures. Application in 1H NMR Metabolomics. *Anal Chem*, **78**, 4281-90.

Hurbet,L. and Arabie,P. (1985) Comparing partitions. *J Classif* **2**(1), 193-218.

Sammon,J.W. (1969) A non-linear mapping for data structure analysis. *IEEE Trans. Comput.*, **C-18**, 401-9.

Shannon,C.E. (1948) A mathematical theory of communication. *Bell Syst Tech J*, **27**(3), 379-423.
